# Angle-Independent Color Change in Thermoresponsive Gel-Immobilized Colloidal Amorphous Film Attached to PET Substrate

**DOI:** 10.3390/polym15244661

**Published:** 2023-12-10

**Authors:** Sato Nakagawa, Yuna Hirano, Mikako Tanaka, Toshimitsu Kanai

**Affiliations:** Graduate School of Engineering Science, Yokohama National University, 79-5 Tokiwadai, Hodogaya-ku, Yokohama 240-8501, Japan; satoshiba310@gmail.com (S.N.); hirano-yuna-gc@ynu.jp (Y.H.); tanaka-mikako-hw@ynu.ac.jp (M.T.)

**Keywords:** stimuli-responsive gel, structural color, colloidal amorphous structure, sensor, film

## Abstract

Gel-immobilized colloidal amorphous structures comprise short-range-ordered monodisperse submicrometer particles embedded into a soft polymer gel. They exhibit an angle-independent structural color that is tunable in response to external stimuli via a volume change in the gel, which has significant potential for the development of sensors that respond to stimuli via angle-independent color changes. In this study, the amorphous structure of a charged colloidal suspension in water was immobilized in a thermoresponsive poly(*N*-isopropylacrylamide) (PNIPAM) gel film and simultaneously attached to a polyethylene terephthalate (PET) substrate. The gel film exhibited a uniform angle-independent color that changed in response to changes in temperature (i.e., thermosensitivity). Attachment to the PET substrate suppressed changes in the gel film area and film distortion, despite significant volume changes in the gel. Consequently, the degree of thermosensitivity was enhanced. The PET-attached gel-immobilized colloidal amorphous film was easy to handle and had excellent flexibility, allowing it to wrap around the surfaces of curved objects. These features are advantageous for sensor applications.

## 1. Introduction

Nanostructures at the scale of the wavelength of visible light can generate color via the reflection or scattering of a specific wavelength of light. This color is called structural color [1,2,3,4]. In contrast to general dyes and pigments, structural colors have unique properties such as nonfading against ultraviolet (UV) light and color tuning according to structural changes; hence, they are expected to be employed as novel colorants and sensors [5,6,7]. Extensive research has been conducted on the preparation and application of colloidal crystals with artificial structural colors [8,9,10,11,12,13,14]. Colloidal crystals utilize periodically arranged monodisperse submicrometer particles to exhibit vivid reflective colors. As the color or reflected light wavelength changes according to Bragg’s law, various colors can be generated by adjusting the diameter of the colloidal particles, the interparticle distance, and the refractive index of the colloidal crystal. Furthermore, colloidal crystals embedded into stimuli-responsive polymers allow for the on-demand tuning of the interparticle distance and Bragg wavelength via the external deformation of the polymer [15,16,17,18,19,20]. However, the color of the colloidal crystals changes with the viewing angle, which may limit their application.

Recently, colloidal amorphous arrays with angle-independent structural colors have been developed [21,22,23]. These colloidal amorphous arrays, which comprise monodisperse submicrometer particles with a short range order, have received increasing attention. Several excellent preparation methods have been reported, including rapid solvent evaporation from a monodisperse colloidal suspension [24,25,26] and drying of a suspension containing two different sizes of monodisperse particles [27,28,29]. However, in these previously reported colloidal amorphous arrays, the colloidal particles are in contact with each other in the dry state. Consequently, monodisperse particles of different sizes must be prepared to create different colors, which is laborious and time-consuming. 

By contrast, we previously achieved the formation of a colloidal amorphous structure in a charged colloidal suspension, in which monodisperse submicrometer-size particles were separated from each other using a liquid medium (water) [30]. When the electrorepulsion between the charged particles was weakened, such as by the addition of salt, the crystalline structure transformed into an amorphous one with an angle-independent color. This amorphous structure with separated particles has a great practical advantage in that the color can be easily altered by simply changing the particle volume fraction or amount of solvent. Furthermore, we successfully immobilized the colloidal amorphous structure in a stimulus-responsive hydrogel film while maintaining a low particle volume fraction and good optical quality. The angle-independent color could be altered on demand by changing the ethanol concentration of the swelling solvent because the resultant volume change in the gel changed the interparticle distance. Thus, the film has the potential to be used as a sensor for detecting external stimuli using angle-independent color changes. However, there are still several issues for the practical application of this material. For example, the thin wet gel film requires careful handling, and the volume changes in the gel (required to alter the color) were accompanied by a significant reduction in the film area and film distortion.

In this study, we immobilized a charged colloidal amorphous structure with a low particle volume fraction in a thermoresponsive poly(*N*-isopropylacrylamide) (PNIPAM) gel film and simultaneously attached the film to a polyethylene terephthalate (PET) substrate. The PET-attached PNIPAM-immobilized colloidal amorphous film exhibited a uniform angle-independent color that changed in response to changes in temperature. Moreover, owing to the attachment of the film to the PET substrate, the reduction in gel film area and film distortion were suppressed, even during significant gel volume changes. Furthermore, the degree of color change in response to temperature changes, that is, the thermosensitivity, was enhanced. Finally, the attachment of the film to the PET substrate improved the ease of handling and flexibility of the material. These features are advantageous for sensor applications.

## 2. Materials and Methods

An aqueous suspension of monodisperse polystyrene particles with diameters of 160 nm (5016 B, Thermo Fisher Scientific, Waltham, MA, USA) was used to prepare a colloidal amorphous film. The suspension was deionized by adding an ion exchange resin (AG 501-X8(D), Bio-Rad, Hercules, CA, USA) and stirring gently for at least two weeks. The obtained charge-stabilized colloidal crystals were centrifuged to prepare concentrated colloidal crystals. A thermoresponsive *N*-isopropylacrylamide monomer (NIPAM, FUJIFILM Wako Pure Chemical Corp., Tokyo, Japan), *N*,*N*′-methylenebisacrylamide crosslinker (BIS, FUJIFILM Wako Pure Chemical Corp., Tokyo, Japan), and 2,2′-azobis [2-methyl-*N*-(2-hydroxyethyl)propionamide] photoinitiator (VA, FUJIFILM Wako Pure Chemical Corp., Tokyo, Japan) were dissolved in ultrapure water (Milli-Q system, Merck KGaA, Darmstadt, Germany) to prepare the gelation reagent. The concentrated charge-stabilized colloidal crystals, gelation reagent, and ultrapure water were added to a square cuvette such that the concentrations of particles, NIPAM, BIS, and VA were 10.7 vol%, 800 mM, 40 mM, and 0.4 mM, respectively, and the total volume was 0.5 mL. To achieve the amorphous structure, a 10 mM aqueous NaCl solution (FUJIFILM Wako Pure Chemical Corp., Tokyo, Japan) was added to the cuvette in 0.5 μL increments, and the reflection spectrum was measured using a fiber spectrometer (Fastevert S-2630, Soma Optics, Ltd., Tokyo, Japan) after each addition. As the salt concentration increased, the intensity of the Bragg reflection peak from the charge-stabilized colloidal crystals was reduced until an amorphous structure with an angle-independent color was achieved. 

**Figure 1:**
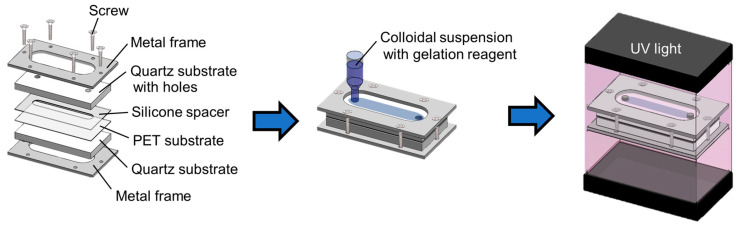
Schematic diagram of the preparation process of the polyethylene terephthalate (PET)-attached poly(*N*-isopropylacrylamide) (PNIPAM)-immobilized colloidal amorphous film.

Figure 1 shows a schematic of the preparation process of the PET-attached PNIPAM-immobilized colloidal amorphous film. The surface of the PET substrate (Lumirror T60, thickness: 100 μm, Toray Industries, Inc., Tokyo, Japan) was treated using plasma ion bombardment (PIB-20, Vacuum Device, Mito, Japan) to attach the gel film to the PET substrate using graft polymerization [31]. The PET substrate was then placed on a quartz substrate along with a silicone separator sheet with a channel (channel height: 0.1 mm; width: 9 mm; length: 50 mm) and quartz substrate with holes and sandwiched within a metal frame. The frame was then fixed in place with screws. 

The colloidal amorphous suspension containing the gelation reagent was bubbled with Ar gas for 5 min and infused into the cell. The suspension was uniformly irradiated with UV light (MBRL-CUV7530, MORITEX Corporation, Saitama, Japan) from the top and bottom surfaces of the cell for 1 h to photopolymerize the gelation reagent. The transmission spectra before and after UV irradiation were measured using a spectrophotometer (V-670, Jasco Corp., Tokyo, Japan) and photographs were taken using a digital camera (IXY Digital 800IS, Canon Inc., Tokyo, Japan). Finally, the cells were disassembled to obtain the PET-attached gel-immobilized colloidal amorphous film. Ultrapure water was placed on the PET-attached gel film and covered with another PET substrate using a 0.3 mm thick silicone spacer.

Further samples were prepared without plasma treatment of the PET substrate. The prepared PNIPAM-immobilized colloidal amorphous film was readily separated from the PET substrate in these samples; therefore, the unattached gel film was sandwiched between two PET substrates along with ultrapure water using a 0.3 mm thick silicone spacer. 

To determine the temperature-dependent changes in the PET-attached and unattached samples, they were placed on a thermal stage, and the transmission spectrum and color were recorded using a spectrophotometer (V-670, Jasco Corp., Tokyo, Japan) and digital camera (IXY Digital 800IS, Canon Inc., Tokyo, Japan), respectively. Changes in the film morphology were also monitored from the digital photographs.

## 3. Results and Discussion

**Figure 2:**
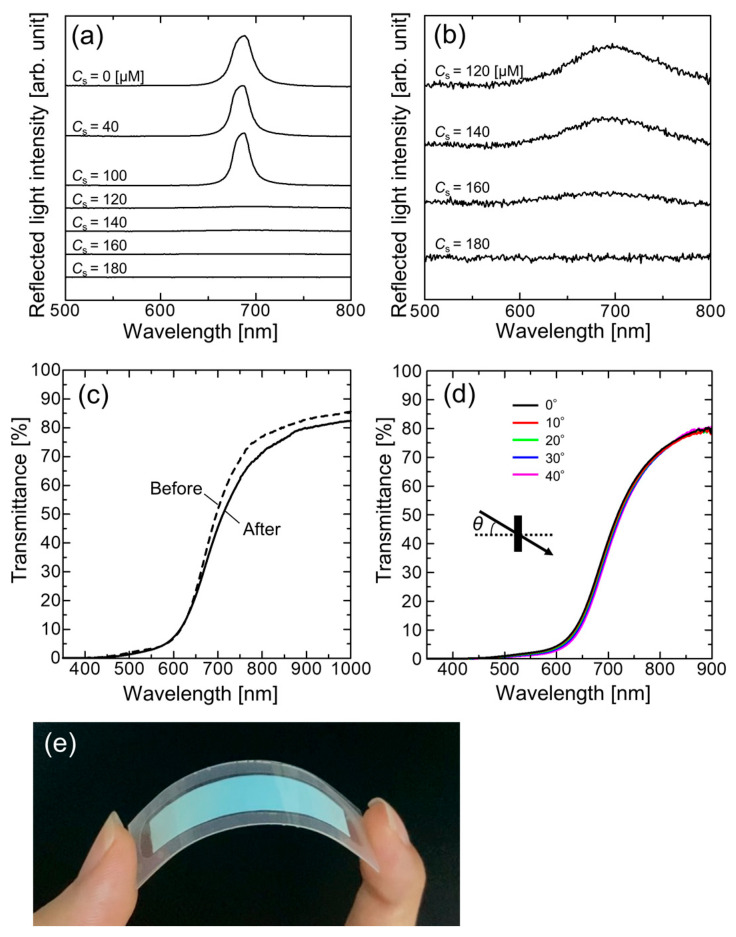
(**a**) Reflection spectra at normal incidence for the colloidal suspensions at different salt concentrations. (**b**) 20-times-magnified reflection spectra at salt concentrations between 120 µM and 180 µM. (**c**) Transmission spectra of colloidal amorphous suspension in the cell before and after ultraviolet (UV) light irradiation. (**d**) Transmission spectra of PET-attached PNIPAM-immobilized colloidal amorphous film at various incident light angles. (**e**) Photograph of PET-attached PNIPAM-immobilized colloidal amorphous film at 31 °C.

Figure 2a demonstrates the change in the reflection spectrum at normal incidence for the colloidal suspension when the salt concentration *C*_s_ was increased. A strong Bragg reflection peak of the charge-stabilized colloidal crystals was observed at approximately 690 nm for the suspensions with *C*_s_ = 0, 40, and 100 µM. Above *C*_s_ = 120 µM, the peak was significantly reduced and only a very small peak owing to the amorphous phase was observed, as shown in the 20× magnified spectra displayed in Figure 2b. The peak was further reduced when the salt concentration was further increased and was not observable above *C*_s_ = 180 µM to transform the random phase.

Figure 2c shows the transmission spectra of the colloidal amorphous suspension in the cell at a salt concentration of 120 μM before and after UV light irradiation. Before UV irradiation, the spectrum was characteristic of the amorphous structure, that is, the transmittance was low in the short-wavelength range and sharply increased at around the Bragg reflection wavelength of the charge-stabilized colloidal crystal with the same particle diameter and volume fraction [30]. A high transmittance of more than 70% was achieved in the longer wavelength range. After UV irradiation, the spectral profile was largely maintained, with a less than 5% decrease in transmittance, which is probably due to the slight deterioration of the amorphous structure. Furthermore, the spectrum was stable upon changing the angle of incident light (Figure 2d). These results indicate that the amorphous structure was successfully immobilized in the PNIPAM gel film. After disassembling the fabricated cell to obtain the PET-attached PNIPAM-immobilized colloidal amorphous film, the sample was easy to handle and had excellent flexibility, as shown in Figure 2e. In contrast to the colloidal crystals, the film showed a uniform color, even on a curved surface.

**Figure 3:**
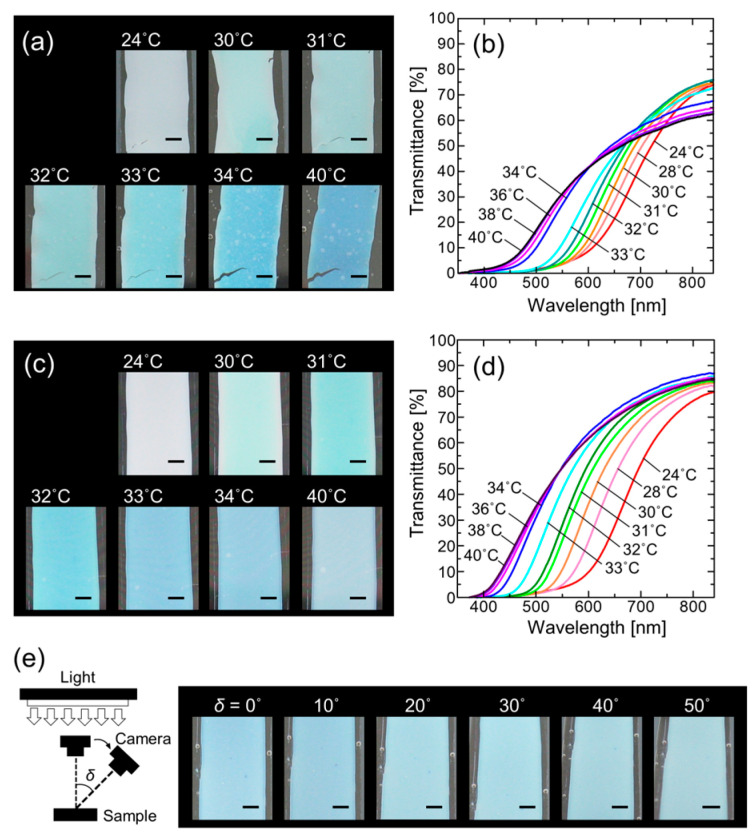
(**a**) Photographs and (**b**) transmission spectra at normal incidence of unattached PNIPAM-immobilized colloidal amorphous film at various temperatures. (**c**) Photographs and (**d**) transmission spectra at normal incidence of PET-attached PNIPAM-immobilized colloidal amorphous film at various temperatures. (**e**) Photographs of PET-attached PNIPAM-immobilized colloidal amorphous film at 33 °C from different camera angles. Scale bars in (**a**,**c**,**e**): 2 mm.

Because the volume of the PNIPAM gel changes significantly at a transition temperature of approximately 32 °C [32], the angle-independent color of the PNIPAM-immobilized colloidal amorphous film can be altered by changing the temperature. The photographs and transmission spectra in Figure 3a,b, respectively, show the temperature-dependent changes in the PNIPAM-immobilized colloidal amorphous film without PET attachment. As the temperature increased, the film shrank, resulting in a color change from light purple at 24 °C to light green at 31 °C and light blue at 34 °C. The colors originate from the coherent scattering of light, and the color change was mainly derived from the reduction in the interparticle distance of the colloidal amorphous structure as the PNIPAM gel in which it was embedded shrank. However, the film distorted and tore during shrinkage. As the temperature increased, the transmission spectrum shifted to shorter wavelengths and the transmittance intensity in the long-wavelength range decreased. The decrease in transmittance in the long-wavelength range was probably caused by the turbulence of the amorphous structure and increased area density of the particles upon gel shrinkage.

By contrast, the PET-attached PNIPAM-immobilized colloidal amorphous film did not exhibit a reduction in the film area without waving or warping as the temperature increased, and the film shrank in the thickness direction. Therefore, it exhibited a more uniform and strong color change (Figure 3c). This was clearly observed in the transmission spectra; the degree to which the spectrum of the PET-attached gel-immobilized colloidal amorphous film blue-shifted was greater than that of the unattached film (Figure 3d). In addition, attachment to the PET substrate preserved the excellent transmission properties, that is, high transmittance in the long-wavelength range, during the temperature change. The enhancement in thermosensitivity is probably caused by an increase in shrinkage in the thickness direction of the PNIPAM gel film, which is complementarily caused by the suppression of in-plane shrinkage [33,34]. Such preservation of the transmittance in the long-wavelength range suggests the amorphous ordering and particle area density are not deteriorated in the in-plane-shrunken PET-attached gel film.

Notably, the PET-attached PNIPAM-immobilized colloidal amorphous film exhibited a uniform angle-independent color at each temperature. For instance, the film appeared a uniform light blue color at 33 °C, regardless of the viewing angle, as shown in Figure 3e.

## 4. Conclusions

A charged colloidal amorphous structure suspended in water was successfully immobilized in a thermoresponsive PNIPAM gel film and simultaneously attached to a PET substrate via photopolymerization. The PNIPAM-immobilized colloidal amorphous film with a low particle volume fraction exhibited a uniform angle-independent color that changed in response to temperature changes. Attachment to the PET substrate suppressed the reduction in film area and film distortion, even when the gel underwent high volume changes. Furthermore, the thermosensitivity was enhanced by an increase in shrinkage in the thickness direction of the PET-attached PNIPAM gel film. The thermosensitivity was controlled with concentrations of monomer and colloids, similar to that of gel-immobilized colloidal crystals. The PET-attached gel-immobilized colloidal amorphous film was easy to handle and had excellent flexibility, allowing its attachment to curved surfaces for sensing applications. These features are advantageous for sensor applications.

## Data Availability

The data presented in this study are available upon request from the corresponding author.
